# Development And Validation of An RNA Binding Protein-Associated Prognostic Model for Colon Adenocarcinoma

**DOI:** 10.7150/jca.103477

**Published:** 2025-05-18

**Authors:** Xiajing Yu, Daixin Guo, Jie Gao, Jialing Hu, Wenyige Zhang, Qijun Yang, Jingyi Wang, Yingcheng He, Kaili Liao, Xiaozhong Wang

**Affiliations:** 1 Jiangxi Province Key Laboratory of Immunology and Inflammation, Jiangxi Provincial Clinical Research Center for Laboratory Medicine, Department of Clinical Laboratory, The Second Affiliated Hospital, Jiangxi Medical College, Nanchang University, Nanchang, Jiangxi, China.; 2 School of Public Health, Jiangxi Medical College, Nanchang University, Nanchang, Jiangxi, China.; 3 The 2nd affiliated hospital, Jiangxi Medical College, Nanchang University, Nanchang, Jiangxi, China.; 4 Department of Anesthesiology, the 2nd affiliated hospital, Jiangxi Medical College, Nanchang University, Nanchang, Jiangxi, China.; 5 Queen Mary College, Jiangxi Medical College, Nanchang University, Nanchang, Jiangxi, China.

**Keywords:** bioinformatics, Colon adenocarcinoma, RNA binding protein.

## Abstract

**Purpose:** We aimed to identify prognostic RNA-binding proteins (RBP) in colon cancer, analyze their biological functions, and develop predictive models for patient prognosis.

**Materials and Methods:** We downloaded COAD's RNA sequencing data from the Cancer Genome Atlas (TCGA) database, and the expression and prognostic value of these RBPs in COAD were systematically evaluated. Differential expression, KEGG, and GO enrichment analyses were then performed. Cytoscape was used to visualize the protein-protein interaction network, and Cox regression was used to establish a predictive model. Finally, the expression of RBP was verified by the HPA database and immunohistochemical staining.

**Results:** A total of 472 differentially expressed RBPs were detected, including 321 up-regulated RBPs and 151 down-regulated RBPs. Four RBPs (MSI2, EZH2, NCL, TERT) were identified as key prognostic genes and used to construct prognostic models, based on this model, the overall survival (OS) of patients in high-risk subgroup was worse than that of patients in the low-risk subgroup. The area under the curve of time-dependent receiver operator characteristic curve of TCGA training set and Gene Expression Omnibus (GEO) validation set was 0.607 and 0.638 respectively, which confirmed that the prognosis model was good, it showed a good ability to identify COAD.

**Conclusion:** In general, our prognostic model is based on 4 RBPs encoding genes, which greatly reduces the cost of sequencing and is more conducive to clinical applications.

## Introduction

COAD is one of the most common causes of cancer-related mortality worldwide^1^. From a genetic point of view, COAD is one of the most well-known cancers. Although the diagnosis and treatment methods have made great progress in the past decades, the treatment of colon cancer is still not satisfactory^2^. The total incidence rate is about 5%, and the 5-year survival rate is 40-60%^3^. The mechanism of occurrence and development of colon cancer has attracted much attention in recent years due to its high mortality and poor prognosis^4^. However, the etiology and pathogenesis of colon cancer are still unclear 5. Therefore, further understanding of the molecular mechanism of COAD and developing effective early screening and diagnosis methods is the key to improving the therapeutic effect and the quality of life of patients. RNA binding protein or microRNAs mediated post-transcriptional regulation together with targeting multiple genes is expected to be involved in the occurrence and development of colon cancer^2^.

So far, more than 1500 RNA-binding proteins (RBP) genes have been identified through genome-wide screening of the human genome^6^. RBPs play a crucial role in post-transcriptional regulation of gene expression, regulating various aspects of RNA metabolism and function, such as polyadenylation, RNA splicing, translocation, stability, and translation. Therefore, they represent the key mechanism of gene regulation in mammalian cells^7^, ^8^. RBPs are involved in the expression of various genes that regulate biological processes and cell functions^9^, ^10^. Many reports have revealed that RBPs are abnormal expressions in tumors, which affect the transformation of mRNA into protein and participate in the carcinogenesis process^11^. Among them, only a few RBPs have been deeply studied and found to play a key role in human cancer. The research on the system function of RBPs will help us to fully understand its role in cancer.

We downloaded the COAD RNA sequencing and clinicopathological data from the Cancer Genome Atlas (TCGA) database. Then, we identified the RBPs that were abnormally expressed between tumor and normal samples by high-throughput bioinformatics analysis and systematically explored their potential functions and molecular mechanisms. Our study identified some RBPs related to COAD, These RBPs can help us understand the molecular mechanism of colon cancer progression. These RBPs may provide potential biomarkers for diagnosis and prognosis.

## Materials and Methods

### Data acquisition

#### TCGA and GEO database analyses

We found and downloaded the corresponding transcriptome data of colon cancer from TCGA, sorted and classified the transcriptome data, transformed the gene ID, downloaded the clinical information of the corresponding patients, extracted the clinical information of age, gender, clinical stage, TNM stage and so on, RBP protein expression was extracted. According to the prognosis and clinical data of patients, the appropriate gene chip was selected from the geodatabase. The chip data was downloaded and annotated. The gene difference was analyzed by limma R package.

The following inclusion criteria were applied: (a) the cancer type is COAD; (b) with prognostic information, including survival time and survival status; (c) the white race; (d) Data are published within 10 years to ensure timeliness and comparability of data; and (e) have complete transcriptome data including RBP gene expression information.

Exclusion criteria included: (a) presence of non-adenocarcinoma types of colon cancer; (b) diagnosis of rectal cancer or other malignancies.

#### KEGG pathway and GO enrichment analyses

The main signal pathways and biological processes of different gene enrichments were studied using the David database (https://david.ncifcrf.gov/). Biological functions of RBPs with different expressions were comprehensively detected. Go analysis included cell composition (CC), molecular function (MF), and biological process (BP), the difference was statistically significant (P < 0.05).

### Protein-protein interaction (PPI) network construction

The protein-protein interaction analysis was carried out by using a string online website, and the obtained protein-protein interaction data was visualized by using Cytoscape software. In addition, the obtained protein-protein interaction network module diagram was divided into several subsets according to the degree of protein-protein interaction. The sub-networks of these sub-datasets were constructed, and the GO and KEGG enrichment analysis and protein-protein interaction analysis were further visualized by Cytoscape software. The difference was statistically significant (P < 0.05).

### Prognostic model construction

The gene expression data and survival time of Gene Expression Omnibus (GEO) chip data and TCGA colon cancer data were combined, and the data after fusion of TCGA and GEO were screened. The prognosis-related RBP genes were screened by using the R package for unicox regression analysis, and the random forest map was drawn, the expression data of the RBP gene in TCGA was used as the training set, and the expression data of the RBP gene in GEO data chip was used as the validation set. The two groups of data were analyzed and plotted by medcale software, and X-tile software 3.6.1 (Yale University, New Haven, CT, USA), and the cut-off values of RBP gene and risk score were calculated. The Kaplan-Meier method was used to calculate the survival rate. The log-rank test was used to test the survival difference between groups. Cox proportional hazards model was used for univariate and multivariate analysis, Finally, R software 3.3.2 (Institute for Statistics and Mathematics, Vienna, Austria) was used to construct a nomogram model and draw nomograms, with P < 0.05 as statistical difference.

### HPA database verifies the expression of hub RBPs in COAD and normal colon tissues

The expression of four key RBPs in colon cancer tissues and adjacent normal tissues was detected in the tissue module and pathway module by using the human protein atlas (HPA) database.

### Immunohistochemical staining

To further confirm the expression of 4 key genes in COAD, we performed immunohistochemical (IHC) staining. Collected samples of COAD patients undergoing treatment in our hospital. Collect 4 pairs of COAD patients for each key gene, including 4 stages I and III respectively. The samples were embedded in paraffin and incubated with rabbit Polyclonal anti-TERT (ABclonal 1:400, A16625, China), rabbit Polyclonal anti-NCL (Proteintech 1:400, 10556-1-AP, China), rabbit Polyclonal anti-MSI2 (Proteintech 1:400, 10770-1-AP, China), monoclonal anti-EZH2 (Proteintech 1:1000, 66476-1-Ig, China) at 4°C overnight. Then, a secondary antibody coupled to horseradish peroxidase (HRP) (1:1000, 5220-0336/5220-0341; China) was incubated with the sections for 60 min at room temperature, followed by 3,3'-diaminobenzidine (DAB substrate system; 2005289) and hematoxylin staining. Images were taken with an Olympus cx-21 (Japan) magnified ×200. Using the analysis software Image-Pro Plus 6.0 (Media Cybernetics, Inc., Rockville, MD, United States), the same intensity of brown color was selected as the unified standard to judge the positivity of all photos, and each photo was analyzed to obtain the positive cumulative optical density (IOD) and tissue pixel area (AREA). The average optical density IOD/AREA (mean density) was calculated. SPSS 19.0 software was used for independent sample t-tests of the average optical density values obtained from stages I and III of COAD tissues. p < 0.05 was defined as statistically significant.

## Results

### Identification of differently expressed RBPs in COAD patients

**Figure 1:**
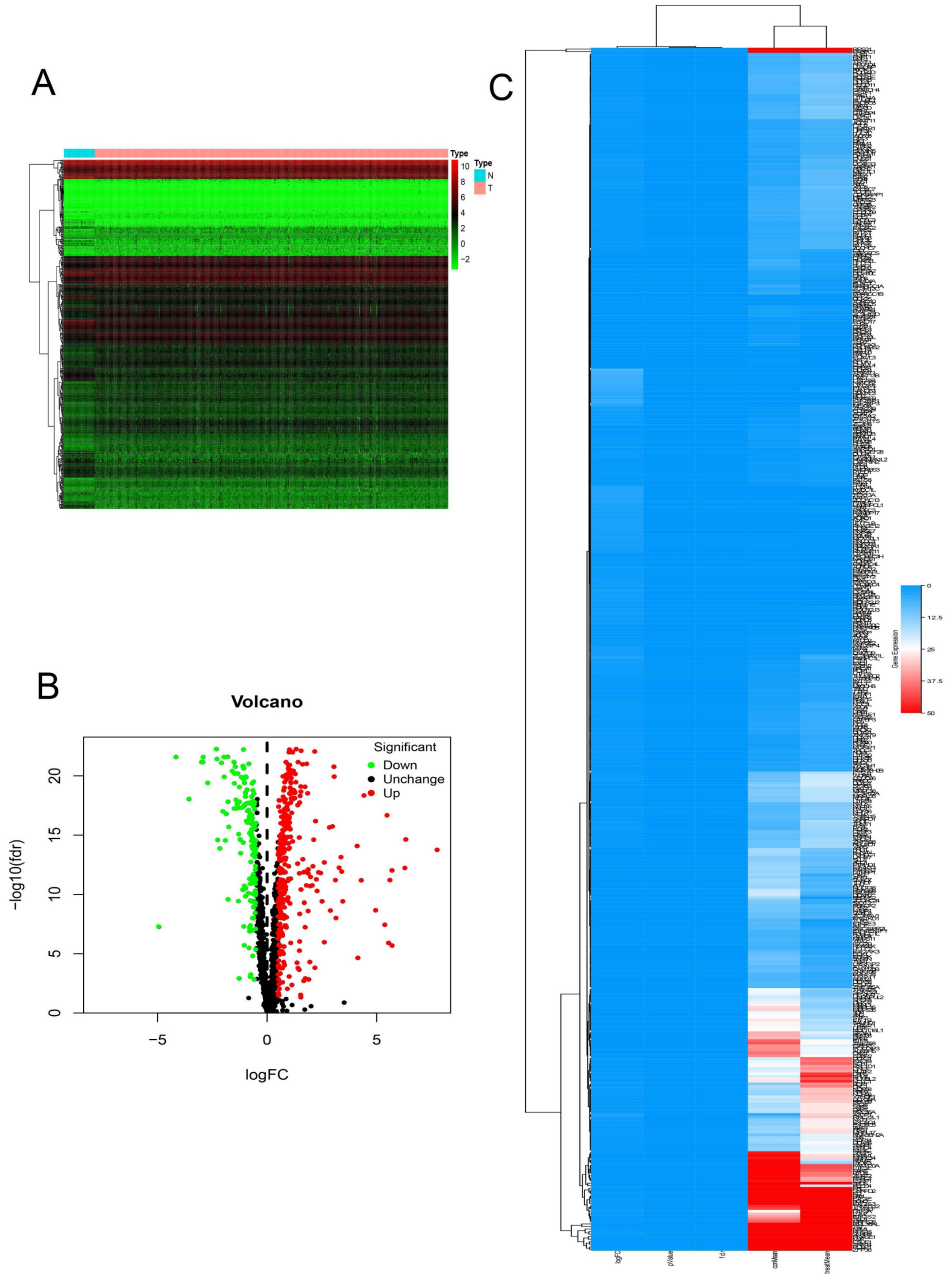
The differentially expressed RBPs in Colon adenocarcinoma.** (A)** Heat map. **(B)** Volcano plot. **(C)** clustering map.

In this study, we systematically analyzed the key role and prognostic value of RBPs in COAD. We downloaded the colon cancer database from TCGA and processed the data with the R software package. A total of 1354 RBPs were included. 472 RBPs met the screening criteria of this study (P < 0.05, | log2FC) | > 1.0), of which 321 were up-regulated, and 151 RBPs were down-regulated. The expression distribution of these RBPs is shown in **(Figure 1)**.

### GO and KEGG pathway enrichment analysis of the differently expressed RBPs

**Figure 2:**
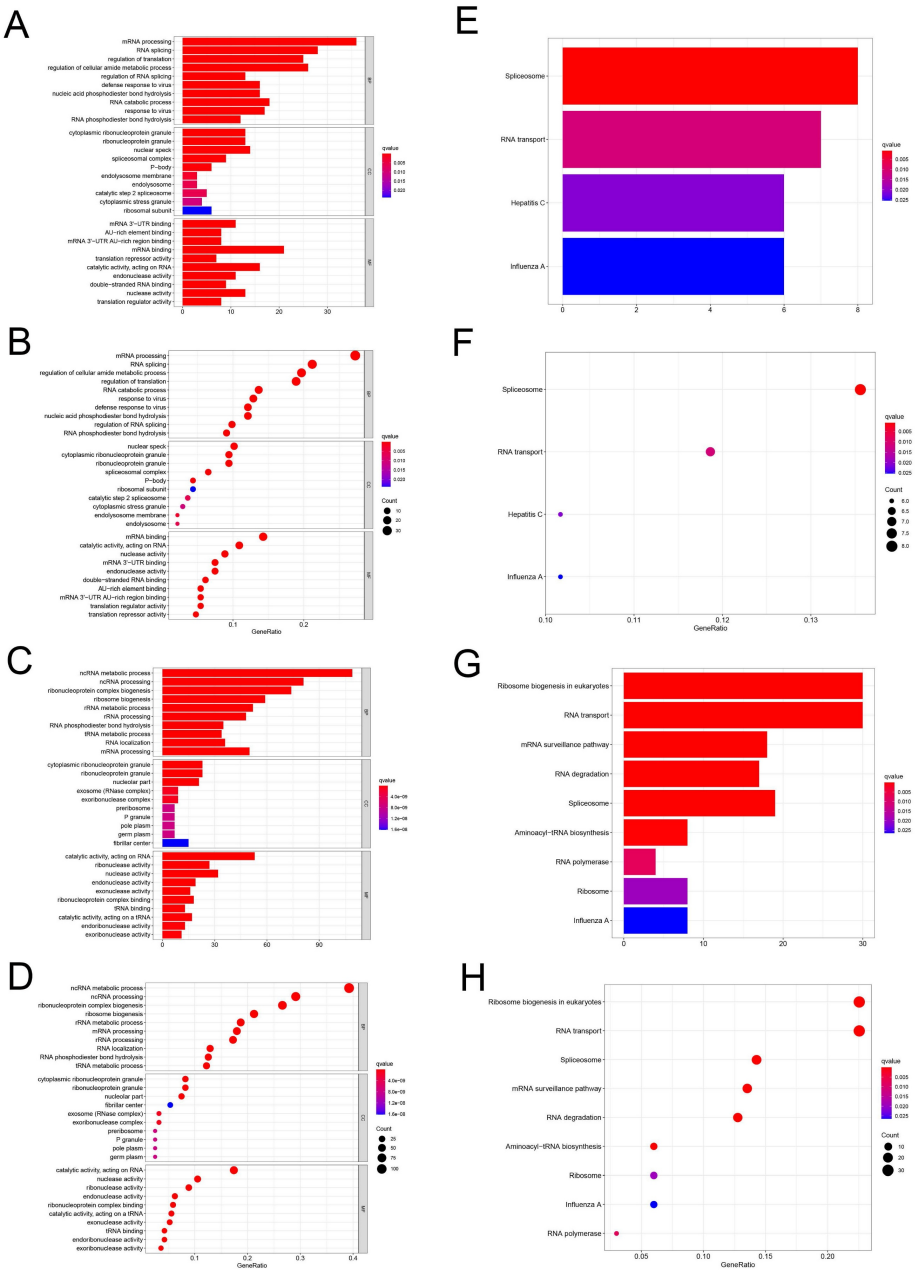
GO and KEGG enrichment analysis. **(A-D)** Use bar and bubble chart to analyze biological processes (BP), molecular functions (MF) and cellular components (CC) in GO. **(E-H)** Bar and bubble chart analysis of KEGG pathway enrichment.

To study the function and mechanism of these identified RBPs, we divided these RBPs into two groups: up-regulated and down-regulated. Then we uploaded these RBPs to the online tool David database for functional enrichment analysis and displayed them with bar graph and bubble graph, Different down-regulated RBPs were significantly enriched in go, which was related to biological process mRNA processing, RNA splicing, regulation of translation, regulation of cellular amide metabolic process, regulation of RNA splicing, defense response to virus, nucleic acid phosphodiester bond hydrolysis, RNA catabolic process, response to virus and RNA phosphodiester bond hydrolysis. The upregulated differently expressed RBPs were significantly enriched in the ncRNA metabolic process, ncRNA processing, ribonucleoprotein complex biogenesis, ribosome biogenesis, rRNA metabolic process, rRNA processing, RNA phosphodiester bond hydrolysis, tRNA metabolic process, RNA localization, mRNA processing **(Figure 2A-D)**. In terms of CC, the decreased differently expressed RBPs were notably enriched in cytoplasmic ribonucleoprotein granule, ribonucleoprotein granule, nuclear speck, spliceosomal complex, P-body, endolysosome membrane, endolysosome, catalytic step 2 spliceosome, cytoplasmic stress granule and ribosomal subunit. While the upregulated differently expressed RBPs were significantly enriched in cytoplasmic ribonucleoprotein granule, ribonucleoprotein granule, nucleolar part, exosome (RNase complex), exoribonuclease complex, pre ribosome, P granule, pole plasm, germplasm, fibrillar center **(Figure 2A-D)**. Through the MF analysis, we found that the decreased differently expressed RBPs were enriched in mRNA 3'-UTR binding, AU-rich element binding, mRNA 3'-UTR AU-rich region binding, mRNA binding, translation repressor activity, catalytic activity, acting on RNA, endonuclease activity, double-stranded RNA binding, nuclease activity, translation regulator activity, and upregulated differently expressed RBPs were mainly enriched in catalytic activity, acting on RNA, ribonuclease activity, nuclease activity, endonuclease activity, exonuclease activity, ribonucleoprotein complex binding, tRNA binding, catalytic activity, acting on a tRNA, endoribonuclease activity, exoribonuclease activity** (Figure 2A-D)**. Moreover, we found that downregulated differently expressed RBPs in KEGG were mainly enriched in Spliceosome, RNA transport, Hepatitis C, and Influenza. A while upregulated RBPs were significantly enriched for Ribosome biogenesis in, eukaryotes, RNA transport, mRNA surveillance pathway, RNA degradation, Spliceosome, Aminoacyl-tRNA biosynthesis, RNA polymerase, Ribosome, Influenza A **(Figure 2E-H)**.

### PPI network construction and key modules selecting

**Figure 3:**
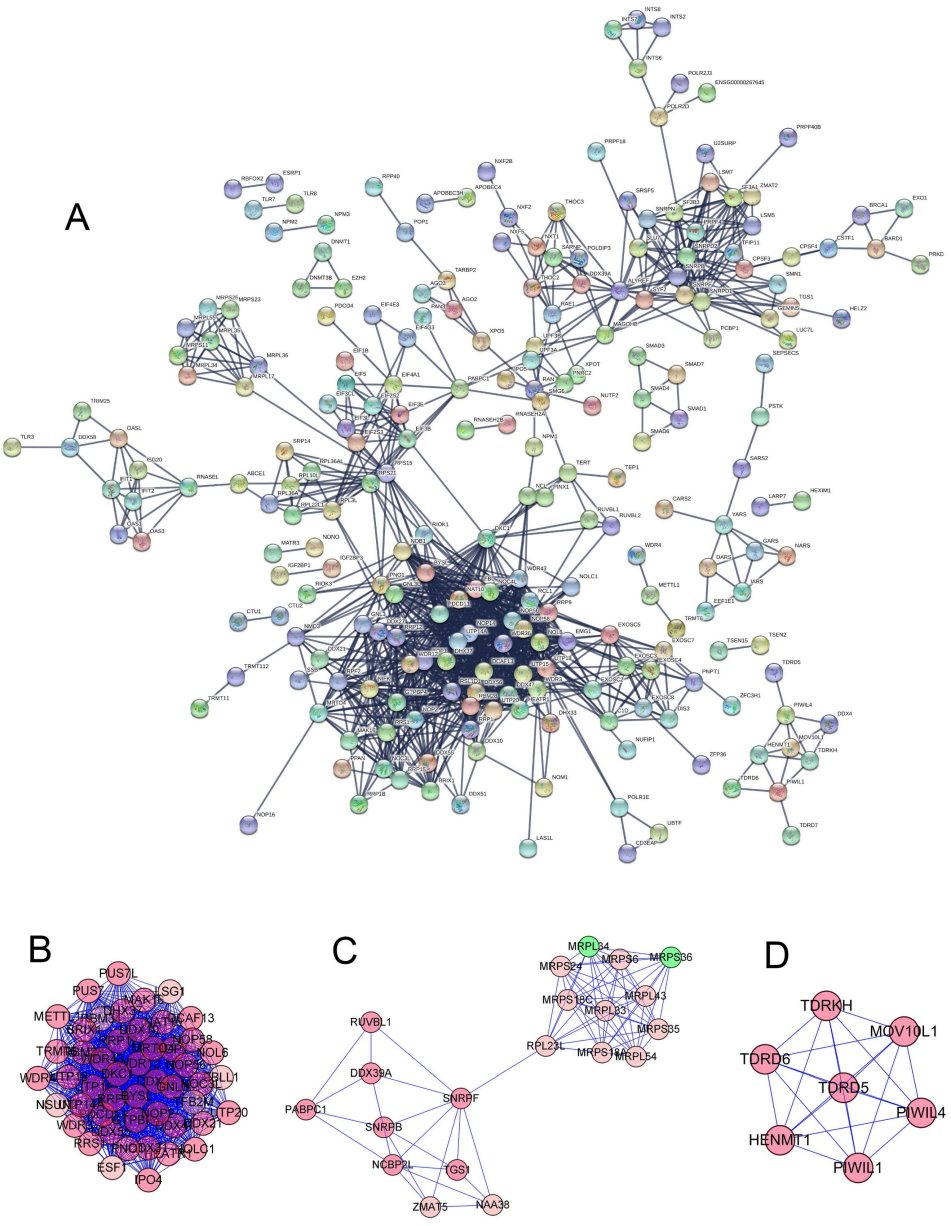
** (A)** PPI network constructed by STRING database. **(B-D)** Subdataset constructed by preCyto. **(E-G)** Subdataset constructed by cytoscape.

**Figure 4:**
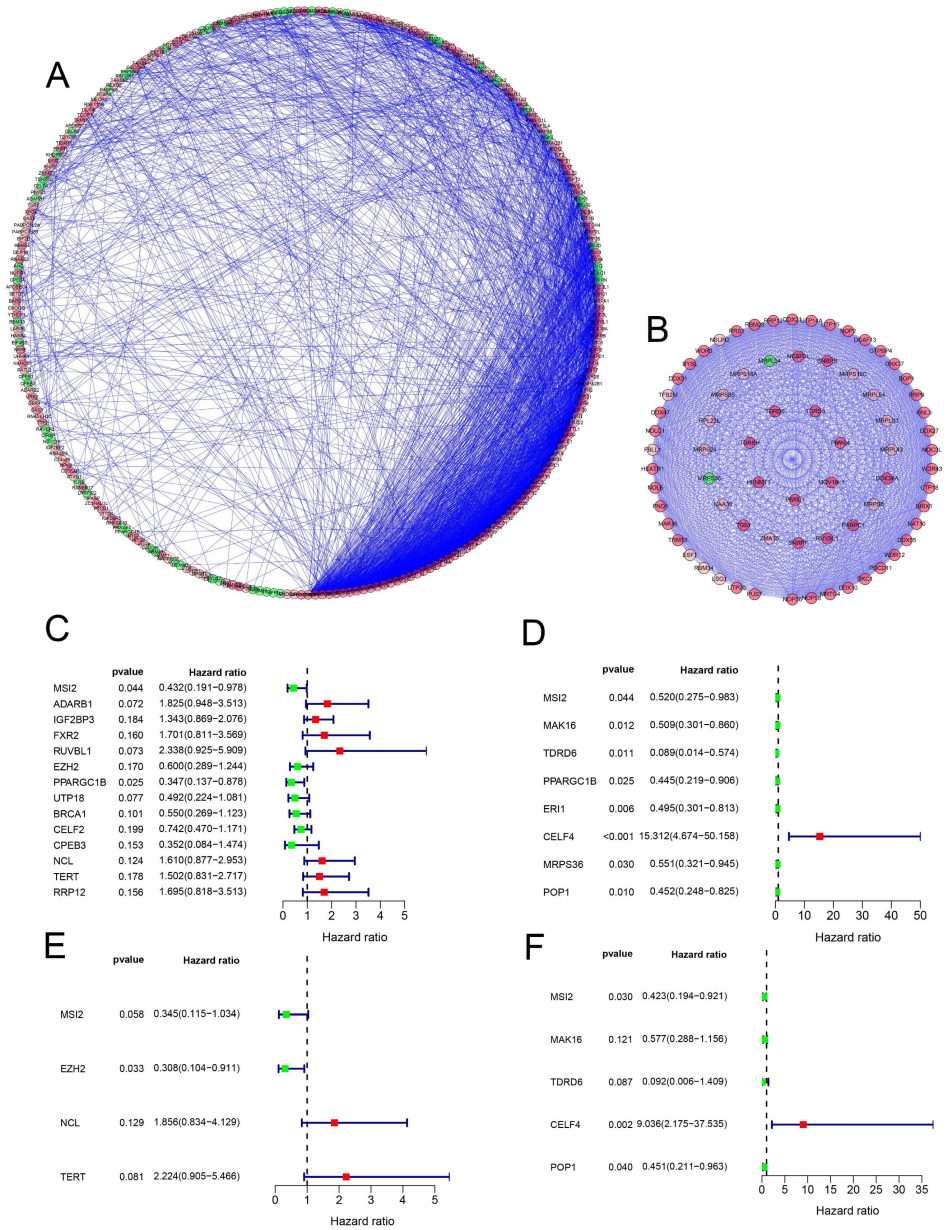
** (A-B)** Protein-protein interaction network, module analysis. **(C-D)** Cox regression analysis: Univariate Cox regression analysis is used to identify the hub RBP in the training data set and the validation data set. **(E-F)** Multivariate Cox regression analysis determines the prognostic-related RBP in the training data set and the validation data set.

To further study the role of different RNA binding proteins in COAD, we created PPI. Based on the data of the string database, we used Precyto and Cytoscape software and visualized the sub-network diagram, including 114 nodes and 1123 edges **(Figure 3A)**. Sub-data set 1 includes 45 nodes and 954 edges **(Figure 3B, E)**. Sub-data set 2 includes 20 nodes and 77 edges **(Figure 3C, F)** Sub dataset 3 consists of 7 nodes and 21 edges **(Figure 3D, G)**. The synthesis of the key three sub-datasets is shown in **(Figure 4A, B)**. These RBPs are abundant in many important pathways, such as ribosomal biogenesis, ribosomal RNA processing, ncRNA processing, piRNA metabolism, DNA methylation and gametogenesis, DNA alkylation, mitochondrial translation extension, termination, and so on.

### Prognosis-related RBPs selecting

A total of 472 RBPs with different expressions were identified from the PPI network. In order to increase the reliability of the results, we used the expression data of the RBP gene in TCGA as the data training set, and the expression data of the RBP gene in the GEO data chip as the data validation set **(Table 1)** to find the molecules that have the greatest impact on the prognosis, 14 and 8 candidate RBPs **(Figure 4C, D)** were obtained respectively, then, multivariate Cox regression analysis was used to identify 4 and 5 key RBPs as independent predictors of COAD **(Figure 4E, F) (Table 2)**.

### Prognosis-related genetic risk score model construction and analysis

**Figure 5:**
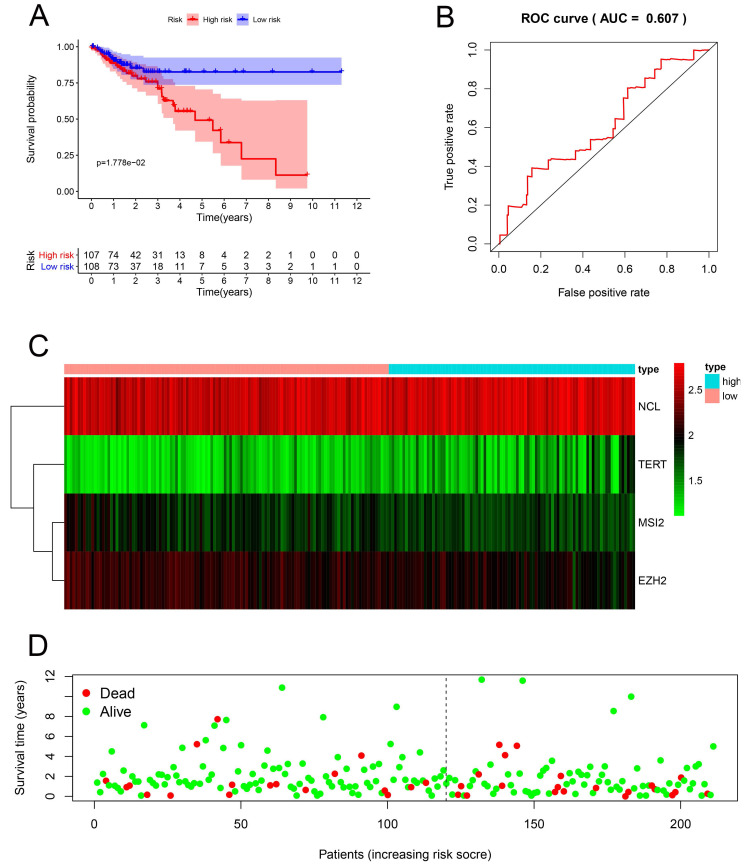
Risk score analysis of the prognostic models of 4 key genes in the TCGA cohort. **(A)** Survival curve of low- and high-risk subgroups. **(B)** ROC curve for predicting OS based on risk score. **(C-D)** expression of heat map, risk score distribution and survival status.

**Figure 6:**
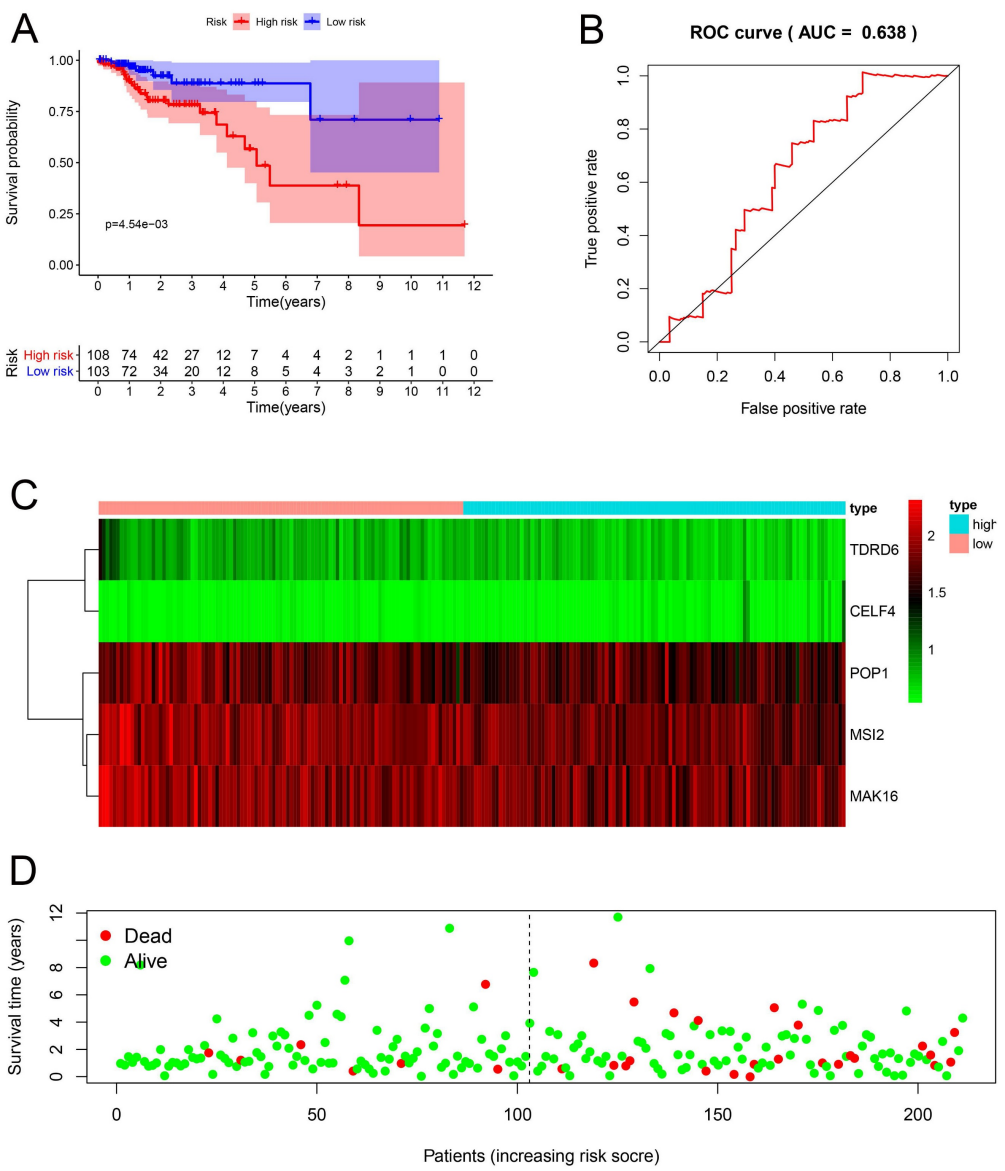
Risk score analysis of the prognostic models of 4 key genes in the GEO cohort. **(A)** Survival curve of low- and high-risk subgroups. **(B)** ROC curve for predicting OS based on risk score. **(C-D)** expression of heat map, risk score distribution and survival status.

Multiple Cox regression analysis was used to identify the key RBPs. Survival analysis was performed to evaluate the predictive ability. Patients with COAD were divided into low-risk subgroups and high-risk subgroup according to the median risk score. The results of the TCGA training set showed that the overall survival rate of the high-risk subgroups was lower than that of the low-risk subgroup **(Figure 5A)**, We performed a time-dependent ROC analysis. We found that the area under the ROC curve (AUC) of the RBPs risk score model was 0.607 **(Figure 5B)**, which had moderate diagnostic performance. The thermograms, survival status, and risk scores of four key RBPs in low and high-risk subgroups are shown in Figure 5C. In addition, we evaluated whether the five key RBPs in the geo-validation set had similar prognostic values in the prediction model. The results showed that the OS of patients with high-risk scores was also worse than that of patients with low-risk scores **(Figure 6A-D)**. These results showed that the prognosis model had good sensitivity and specificity.

### Construction of a nomogram based on the eight-hub RBPs

**Figure 7:**
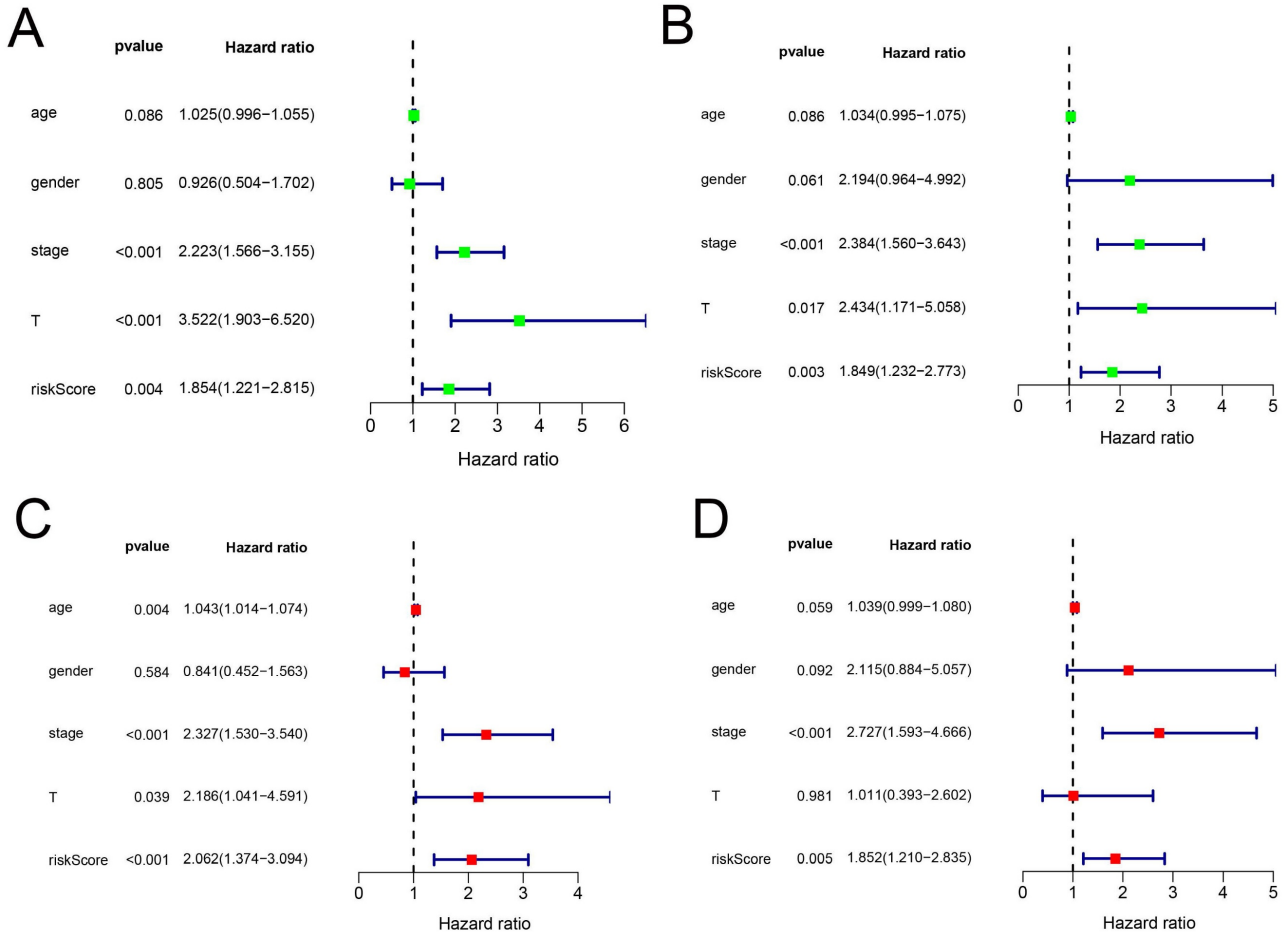
The prognostic value of different clinical parameters. **(A-B)** Clinical parameters of training set and validation set of univariate analysis data. **(C-D)** Multiple factors determine the independent prognostic factors of the data training set and validation set.

**Figure 8:**
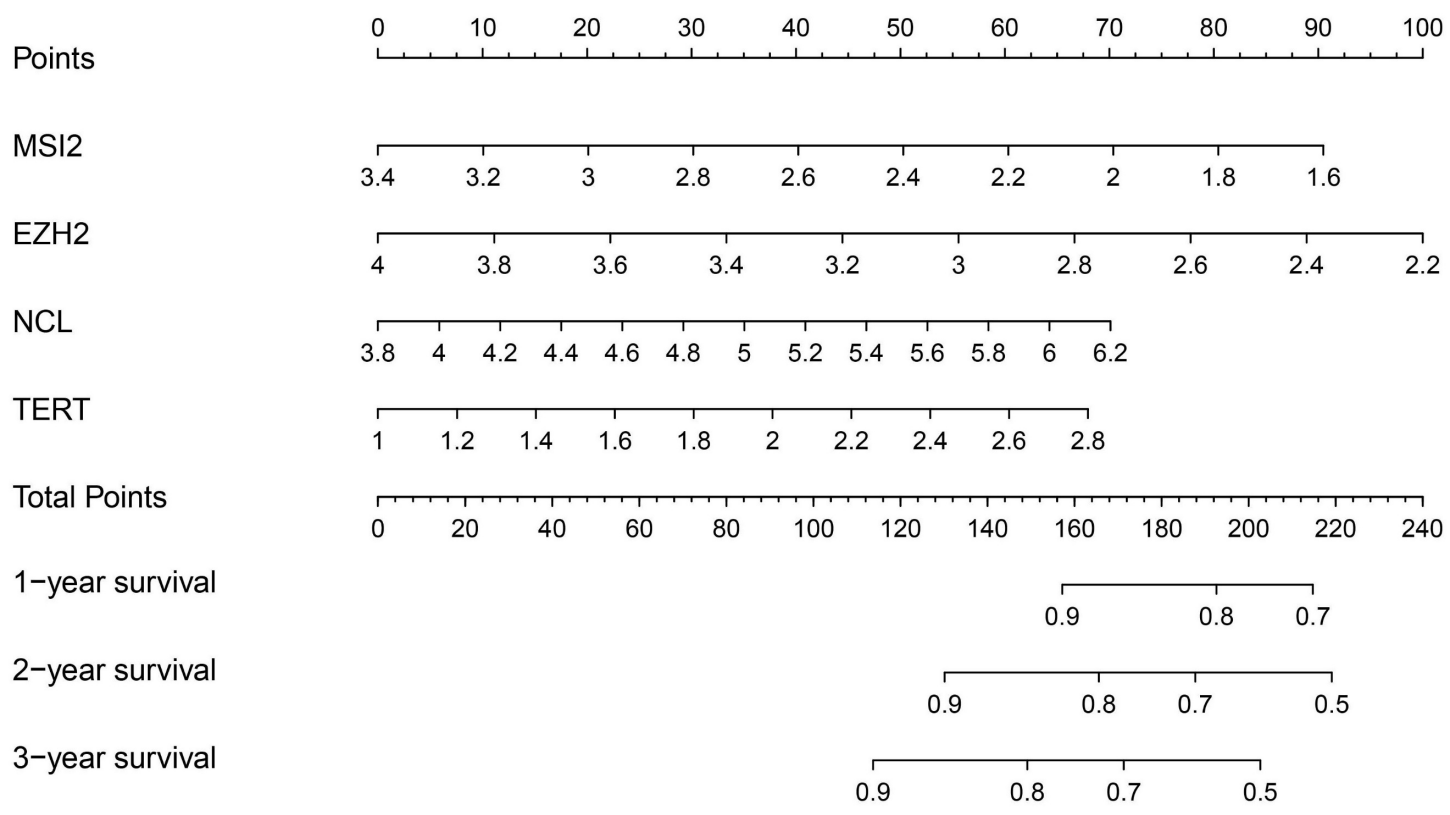
Nomogram prediction method for 1 year, 2 year and 3year OS of COAD patients in the TCGA cohort.

In addition, we evaluated the prognostic significance of different clinical features and TCGA in patients with COAD by Cox regression analysis. The results showed that tumor stage, primary tumor location, and risk score were correlated with the prognosis of patients with colon cancer in the data training set and validation set (P < 0.05)** (Figure 7A, B) (Table 2)**. However, by multivariate regression analysis, we only found that tumor stage and risk score were independent prognostic factors related to the survival rate of colon cancer (P < 0.01) **(Figure 7C, D) (Table 3).** Based on multivariate Cox analysis, we assigned the points to individual variables by the point scale in the nomogram. We drew a horizontal line to determine the points of each variable, added the points of all variables, calculated the total point of each patient, and standardized it to a distribution of 0 to 100. We can draw a vertical line between the total point axis and each prognosis axis to calculate the 1-year, 2-year, and 3-year estimated survival rates of COAD patients, this can help relevant practitioners to make clinical decisions for COAD patients **(Figure 8)**.

### Validation of the prognostic value and expression of hub RBPs from the HPA database

**Figure 9:**
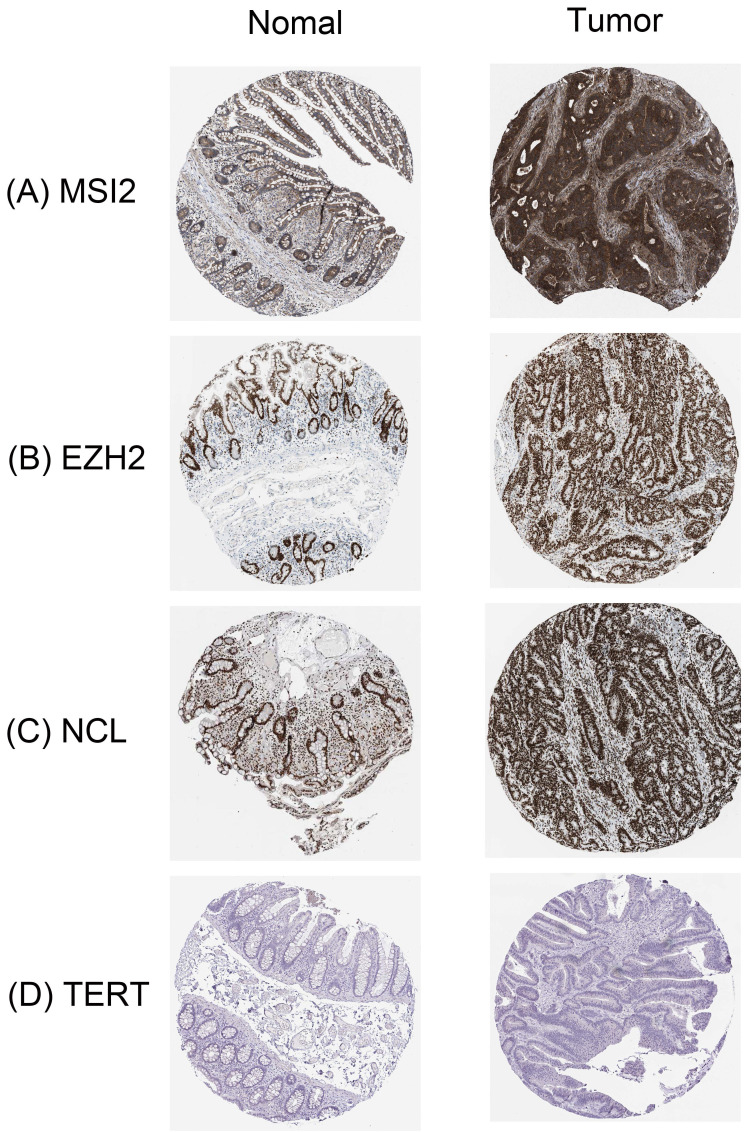
Verification of hub RBPs expression in COAD and Normal colon tissue using the HPA database. **(A)**: MSI2, **(B)**: EZH2, **(C)**: NCL, **(D)**: TERT.

In order to further determine the expression of these key RBPs in COAD, we used the human protein map database to further analyze. Immunohistochemical results showed that compared with normal colon tissues, MSI2, EZH2, and NCL were significantly higher expressed in colon cancer tissues except TERT. In addition, MSI2 was moderately expressed in normal colon tissues, NCL was low expressed, and EZH2 and TERT were not expressed **(Figure 9)**.

### Validation of the prognostic value and expression of hub RBPs from Immunohistochemical Staining

**Figure 10:**
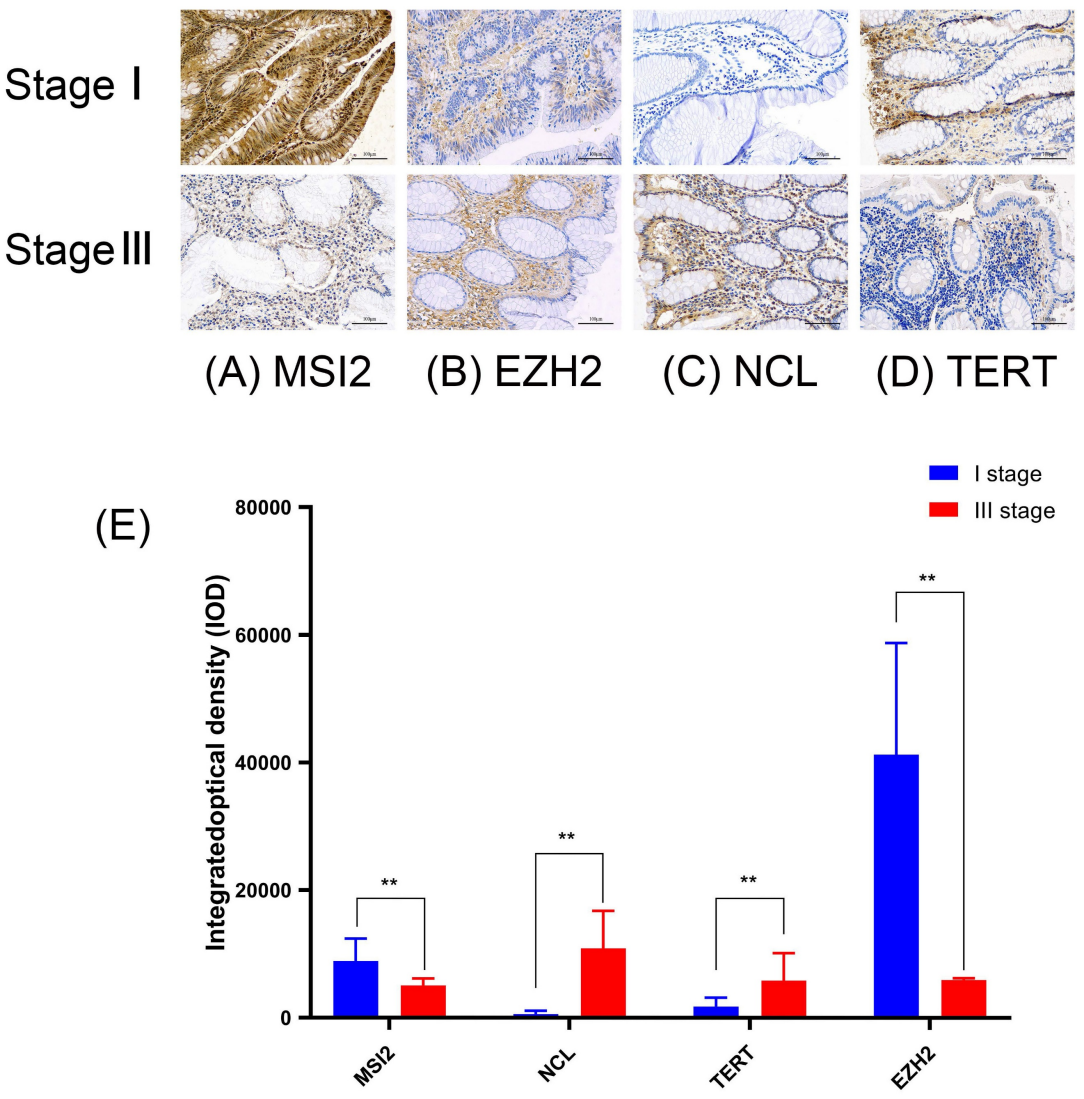
Validation the prognostic value and expression of hub RBPs from Immunohistochemical Staining. Compared with stage I of COAD, NCL **(C)** and TERT **(D)** are slightly higher in stage III of COAD (p˂0.01), MSI2 **(A)** and EZH2 **(B)** are lower expressed (p˂0.01).** (E)** IOD of four key genes in COAD stage I and stage III.

To further verify the reliability of the above results, we performed immunohistochemical (IHC) staining, compared with stage I of COAD, NCL, and TERT are slightly higher in stage III of COAD (p ˂ 0.01), however, MSI2 and EZH2 are lower expressed (p ˂ 0.01). These results are in line with the results of the HPA database **(Figure 10) (Table 4)**.

## Discussion

RBP imbalance has been reported in many malignant tumors 11, 12. However, only a small number of RBPs have been in-depth research, partially confirming that they are involved in the occurrence and development of cancer^13^. In this study, we identified 472 differently expressed RBPs in colon tumor tissues and normal tissues based on the TCGA and GEO databases. We systematically analyzed the relevant biological pathways and constructed the co-expression network and PPI network of these RBPs. In addition, we also performed single-factor Cox regression analysis, survival analysis, multi-factor Cox regression analysis, and ROC analysis on key RBPs to further explore their biological functions and clinical significance. We are based on 4 key RBP genes related to prognosis. A risk model for predicting the prognosis of COAD was constructed. These findings may help develop new biomarkers to diagnose COAD patients and their prognosis.

Functional pathway enrichment analysis shows that different RBPs are present in mRNA processing, RNA splicing, translation regulation, cellular amide metabolism process regulation, RNA splicing regulation, virus defense response, nucleic acid phosphodiester bond hydrolysis, RNA catabolism, viral response, RNA Hydrolysis of phosphodiester bonds and enrichment in ribosomal biogenesis. Studies have confirmed that the regulation of translation, RNA processing, and RNA metabolism is related to the occurrence and development of many human diseases^14^-^16^.

It has recently been shown that NOVA1 knockout can significantly change the TERT transcript of colon cancer^17^. RBP MSI2 has many important roles in some tissues and is also overexpressed in many cancers^18^-^22^. Studies have shown that Aza-9 binds to MSI2, thereby inhibiting the proliferation of colon cancer cell lines, inducing apoptosis and autophagy, and down-regulating Notch and Wnt signaling^23^. In addition, studies report that PRC2 and DDX5-related lncRNA (PRADX) are highly expressed in colon cancer. This finding indicates that PRADX is mainly located in the nucleus of tumor cells and can bind to EZH2 protein through the 5'end sequence, thereby affecting the occurrence of colon cancer^24^. Ribonucleoprotein particles are the key area for performing protein biosynthesis. Changes in ribonucleoprotein affect the translation process and are associated with tumor progression^25^. Umar et al^26^ through chromatin immunoprecipitation showed that EZH2 can reduce WIF1 mRNA and protein expression by occupying the Wnt inhibitor-1 (WIF1) promoter, thereby abnormally regulating the Wnt/β-catenin signaling pathway to promote colonic crypt hyperplasia and tumorigenesis. Studies have shown that the analysis of the KEGG pathway in this study shows that its abnormal expression of RBPs affects ribosome biogenesis, RNA transport, mRNA monitoring pathways, RNA degradation, spliceosome, aminoacyl tRNA biosynthesis, RNA polymerase, etc. in eukaryotes. Significantly enriched to regulate the occurrence and development of colon cancer.

In addition, we established a protein-protein interaction network of these differently expressed RBPs, and obtained a key module containing 72 key RBPs. Among these key RBPs, many have been shown to play an important role in the development and progression of tumors. Although the link between most of the differently expressed RBPs and colon cancer is unclear, some RBPs have been reported to be associated with other tumors. In this study, through the module analysis of the PPI network, COAD is related to mRNA processing, RNA splicing, translation regulation, eukaryotic ribosome biogenesis, and regulation of cellular amide metabolism.

We screened hub RBPs by univariate Cox regression analysis, survival analysis, and multivariate Cox regression analysis. A total of 4 RBPs were identified as key prognostic-related RBPs, including MSI2, EZH2, NCL, and TERT. There have been research reports on MSI2^27^, the expression of TERT^17^ is related to the tumorigenesis and development of colon cancer patients. The high proliferation index of EZH2 in tumors is usually related to the overexpression of EZH2.The absence of EZH2 can inhibit the growth of cancer cells, induce senescence and apoptosis, and reduce invasion and metastasis^28^-^30^. The overexpression of EZH2 and tumor invasion or metastasis has been confirmed. The results of our HPA database study show that EZH2 is highly expressed in colon cancer, which is consistent with our findings. So far, the research of RNA-binding protein NCL in colon cancer has not been reported. Our research results provide useful exploration value and space for the later research of NCL in COAD and help to develop new therapeutic targets and prognostic molecular markers. We use the 4 hub RBPs coding genes in the TCGA cohort training set to establish a risk model to predict the prognosis of COAD through multiple stepwise Cox regression analyses. ROC curve analysis shows that these four gene signatures have good diagnostic capabilities and can screen out COAD patients with poor prognosis. However, the molecular mechanisms of these four RBPs involved in the occurrence of colon cancer are still unclear, and further exploration of the underlying mechanisms may be valuable. Subsequently, we established a nomogram 1-year, 2-year, and 3-year OS prediction method. We also used Kaplan Meierplotter to detect the prognostic value of 4 RBPs encoding genes, and the results were consistent with the prognostic analysis results of the TCGA cohort. These results show that the prognostic model of 4 gene characteristics has a certain value in adjusting the treatment plan of colon cancer patients.

In general, our prognostic model is based on 4 RBPs encoding genes, which greatly reduces the cost of sequencing and is more conducive to clinical applications. In addition, the 4 gene prediction models have a better effect on the survival prediction of COAD patients. In addition, RBPs-related gene signatures show important biological functions, suggesting that they may be used in clinical adjuvant therapy, which is not always the case in previous studies. Nevertheless, this study has some limitations. First, the data in this study mainly originated from TCGA and GEO databases, and the samples in different datasets may be heterogeneous, which in turn affects the accuracy and generalization ability of the results. Second, the research method focused on bioinformatics analysis and lacked direct experimental validation to support the conclusions. Although immunohistochemical staining can detect protein expression levels and provide some experimental basis for the findings, it fails to comprehensively reveal the specific mechanisms of RBPs in colon carcinogenesis. In addition, this study was designed retrospectively, and prospective studies are needed to validate the results. Furthermore, although the predictive model we constructed demonstrated good diagnostic efficacy, it may be affected by multiple factors, such as individual patient differences and differences in treatment regimens, and the accuracy and reliability of the model need to be further validated in diverse patient populations and clinical settings. Finally, the study revealed the association of four key RBPs, MSI2, EZH2, NCL, and TERT, with the prognosis of colon cancer; however, the molecular mechanisms of the involvement of these RBPs in the pathogenesis of colon cancer have not yet been clarified, limiting the in-depthness and practicality of the study. Future studies need to explore the specific mechanisms of the roles of these RBPs in colon carcinogenesis and progression further.

In summary, we systematically explored the expression and prognostic value of differently expressed RBPs in COAD through a series of bioinformatics analyses. These RBPs may be involved in the occurrence, development, invasion, and metastasis of COAD. Four RBPs were constructed. The prognostic model of the coding gene may be an independent prognostic factor of COAD. As far as we know, this is the first report on the development of a prognostic model of COAD related to RBPs. In addition, we found that the key RNA binding protein NCL is in colon cancer tissue Significantly high expression in the two, the study between the two has not been reported. Our research results will help reveal the pathogenesis of COAD, and develop new therapeutic targets and prognostic molecular markers.

## Supplementary Material

Supplementary methods and table.

## Figures and Tables

**Table 1:** Clinic parameters of downloaded colon cancer patients.

	TCGA	GEO
**Gender**		
Male	238(52.7%)	320(55.5%)
Female	214(47.3%)	257(44.5%)
Age	67.1	66.9
**Clinical Stage (TNM )**		
Stage I-II	254(56.2%)	308(53.4%)
Stage III-IV	198(43.8%)	269(46.6%)
Futime	705.07	1493.8578
**Fustat**		
Death	88(19,5%)	182(31.5%)
Alive	364(80.5%)	395(68.5%)

**Table 2:** Four prognosis-associated hub RBPs identified by multivariate Cox regression analysis.

RBP name	Coef	HR	Lower 95% CI	Upper 95% CI	p-value
Data training set					
MSI2	-1.0644	0.3449	0.1150	1.0345	0.0575
EZH2	-1.1764	0.3084	0.1044	0.9107	0.0332
NCL	0.6185	1.8562	0.8344	4.1291	0.1295
TERT	0.7993	2.2240	0.9049	5.4662	0.0815
Data validation set					
MSI2	-0.8605	0.4230	0.1942	0.9213	0.0303
MAK16	-0.5499	0.5770	0.2880	1.1560	0.1209
TDRD6	-2.3895	0.0917	0.0060	1.4088	0.0865
CELF4	2.2012	9.0361	2.1753	37.5347	0.0024
POP1	-0.7969	0.4507	0.2110	0.9629	0.0396

**Table 3:** The prognostic value of different clinical parameters.

	Univariate analysis			Multivariate analysis		
	**HR**	**95%CI**	**P-value**	**HR**	**95%CI**	**P-value**
Age	1.03	1.00-1.05	0.0862	1.04	1.01-1.07	0.0037
Gender	0.93	0.50-1.70	0.8052	0.84	0.45-1.56	0.5840
Stage	2.22	1.57-3.15	0.0000	2.33	1.53-3.54	0.0001
T	3.52	1.90-6.52	0.0001	2.19	1.04-4.59	0.0388
Risk Score	1.85	1.22-2.82	0.0038	2.06	1.37-3.09	0.0005

**Table 4:** Average optical density of Four key RBP in COAD.

Cancer	MSI2	NCL	TERT	EZH2
Mean density	Mean ± SD	Mean ± SD	Mean ± SD	Mean ± SD
Stage I	8879.7±3534.694**	579.18±521.29.6	1785.9±1381.409	41288±17484.76**
Stage III	5059.3±1112.422	10890±5878.178**	5845.3±4320.255**	5953.6±290.3075

A p-value < 0.05 was considered statistically significant. The symbols “**” refer to p-values < 0.01.

## Abbreviations

RBPs: RNA binding proteins
COAD: Colon adenocarcinoma
OS: overall survival
TCGA: the Cancer Genome Atlas
CC: cell composition
MF: molecular function
BP: biological process
PPI: Protein-protein interaction
GEO: Gene Expression Omnibus
HPA: human protein atlas
IHC: Immunohistochemical
