# Supplementary material for: Development And Validation of An RNA Binding Protein-Associated Prognostic Model for Colon Adenocarcinoma
Source: J Cancer. 2025 May 18;16(8):2537–52. doi: 10.7150/jca.103477 (PMC12170993; doi:10.7150/jca.103477)
Supplement: Supplementary file 1 — Supplementary methods and table. [file jcav16p2537s1.pdf]

The detailed protocol and criteria of Immunohistochemistry

1. Experimental Equipment and Reagents

1.1 Experimental Equipment

Name	Manufacturer	Model
Dehydrator	Wuhan Junjie Electronic Co., Ltd.	JJ-12J
Embedding Machine	Wuhan Junjie Electronic Co., Ltd.	JB-P5
Pathological Microtome	Shanghai Leica Instruments Co., Ltd.	RM2016
Freezing Platform	Wuhan Junjie Electronic Co., Ltd.	JB-L5
Tissue Spreader	Jinhua Kedi Instrument and Equipment Co., Ltd., Zhejiang Province	KD-P
Oven	Shanghai Huitai Instrument Manufacturing Co., Ltd.	DHG-9140A
Slide	Jiangsu Shitai Experimental Equipment Co., Ltd.	
Cover Glass	Jiangsu Shitai Experimental Equipment Co., Ltd.	10212432C
Microwave Oven	Galanz Microwave Oven Appliance Co., Ltd.	P70D20TL-P4
Decolorizing Shaker	Beijing Liuyi Instrument Factory	WD-9405A
Vortex Mixer	Tianyue Electronics	TYXH-II
Pipette	Dalong	KE0003087/KA0056573

1.2 Main Experimental Reagents

Reagent	Manufacturer	Product Code	Dilution Ratio
Absolute Ethanol	Sinopharm Chemical Reagent Co., Ltd.		
Xylene	Sinopharm Chemical Reagent Co., Ltd.		
EDTA (PH8.0) Antigen Repair Solution	Wuhan Baiqiandu Biology	B2001	

EDTA (PH9.0) Antigen Repair Solution	Wuhan Baiqiandu Biology	B2002	
Citric Acid (PH6.0) Antigen Repair Solution	Wuhan Baiqiandu Biology	B2010	
PBS Buffer	Wuhan Baiqiandu Biology	B0002	
Tyramide-Biotin	Wuhan Baiqiandu Biology		1/1000
Streptavidin-HRP	Wuhan Baiqiandu Biology		1/1000

1.3 Primary Antibodies

Name	Manufacturer	Product Code	Species	Dilution Ratio	Retrieval Method
TERT	ABclonal	A16625	Rabbit	1:400	High-pressure retrieval
NCL	Sanying	10556-1-AP	Rabbit	1:400	High-pressure retrieval
MSI2	Sanying	10770-1-AP	Rabbit	1:400	High-pressure retrieval
EZH2	Sanying	66476-1-Ig (Monoclonal)	Mouse	1:400	High-pressure retrieval

1.4 Secondary Antibodies

Name	Manufacturer	Product Code	Dilution Ratio
HRP-Labeled Goat Anti-Mouse	SeraCare	5220-0341	1:1000
HRP-Labeled Goat Anti-Rat	SeraCare	5220-0364	1:200

2. Experimental Procedures for Immunohistochemical Staining on Paraffin Sections

Paraffin sections were first immersed in xylene, followed by absolute ethanol, and various concentrations of alcohol to achieve deparaffinization and hydration. Next, antigen retrieval was conducted using a citric acid repair solution in a pressure cooker,

and the sections were washed with PBS. After drying the sections, the edges of the tissues were outlined with a histochemical pen. Then, serum blocking was performed by applying 3% BSA. Following the removal of the blocking solution, the primary antibody was added and incubated overnight at 4°C in a wet box. The next day, the sections were washed with PBST, and a secondary antibody (labeled with HRP) was applied, followed by incubation at room temperature in the dark. DAB staining was then performed, along with hematoxylin counterstaining of the nuclei. Finally, the sections were dehydrated with absolute ethanol, treated with xylene for transparency, and sealed with neutral gum.

3. Interpretation of Immunohistochemical Results

The nuclei stained with hematoxylin appear blue under a white light microscope, and positive expression appears as corresponding brown or brownish-yellow.

3.1 Staining intensity:

Ative (-): There is no positive staining or only very weak non-specific background staining in the section.
Weak Positive (+): There was positive staining in the sections, but the staining was light and unevenly distributed.
Moderate positive (++): there was significant positive staining in the sections, with moderate intensity and relatively uniform distribution.
Strong positive (+++): There is strong positive staining in the section, which is deep and evenly distributed, usually covering most or all of the tissue area.

3.2 Proportion of positive cells:

Low proportion: <10% of cells showed positive staining.
Moderate proportion: between 10% and 50% of cells showed positive staining.
High proportion: >50% of cells showed positive staining

4. Analysis of Immunohistochemical Integrated Optical Density (IOD)

Randomly select at least 3 fields of view at 200x magnification from each section within each group for photography. Ensure that the tissue fills the entire field of view during photography and that the background light is consistent for each photo. Use Image-Pro Plus 6.0 software to select the same brownish-yellow color as the unified criterion for judging positivity in all photos. Analyze each photo to obtain the integrated optical density (IOD) and tissue pixel area (AREA) of the positive staining. Calculate the average optical density as IOD/AREA (average density).

Supplemental Table S1. The case number for the Immunohistochemistry

Cancer	Stage	The Case Number
EZH2	Stage I	1126529
		1131422
		1150530
	Stage III	1157199
		1181859
		1109498
MSI2	Stage I	1114761
		1142417
		1146087
	Stage III	1148450
		1173759
		1180042
		1189434
NCL	Stage I	1114764
		1145042
		1173023
		1194299
	Stage III	1129799
		1142763
		1171325
		1191914
TERT	Stage I	1061387
		1098217
		1144723
		1169722
	Stage III	1135343
		1180298
		1195076
		1209080
